# The role of Ashwagandha in modulating gut parameters in dogs—a randomized double-blind placebo-controlled trial

**DOI:** 10.3389/fvets.2024.1491989

**Published:** 2025-01-22

**Authors:** Kala Kumar Bharani, Ashok Kumar Devarasetti, Rajendar Bobbili, Amit Khurana, Donga Durga Veera Hanuman, Roupesh Gudepu, Swapna Guda

**Affiliations:** ^1^Department of Pharmacology and Toxicology, School of Veterinary and Animal Sciences, Centurion University of Technology and Management, Paralakhemundi, India; ^2^Department of Veterinary Biochemistry, College of Veterinary Science (CVSc), Warangal, India; ^3^Department of Veterinary Pharmacology and Toxicology, College of Veterinary Science (CVSc), Hyderabad, India; ^4^Department of Animal Nutrition, Nagpur Veterinary College, Maharashtra Animal and Fishery Sciences University, Nagpur, India

**Keywords:** adaptogen, aging-related changes, healthy gut, microbiome, Ashwagandha root extract, geriatric dogs

## Abstract

**Introduction:**

This study explored the role of *Withania somnifera*/Ashwagandha root extract (ARE) on important gut–microbiome parameters in healthy geriatric dogs. We hypothesized that ARE might promote a healthy gut by its adaptogenic and anti-inflammatory effects and improve vital parameters for healthy ageing.

**Methods:**

A randomized, double-blind, placebo-controlled trial was conducted in Telangana, India. Twelve healthy geriatric Beagle dogs aged 12–15 years were enrolled. The dogs were divided into two groups to receive ARE (15 mg/kg, once daily, orally, for 2 months) or a placebo control. Various parameters were assessed, including serum haematology, biochemical markers, stool parameters, and gut–microbiome parameters.

**Results:**

The erythrocyte counts and haemoglobin levels were significantly increased with ARE (*p* < 0.01 and *p* < 0.001). Moreover, a significant decrease in important serum liver biomarkers (alanine transaminase [ALT], aspartate transaminase [AST]; *p* < 0.01 and *p* < 0.001 at day 60) was observed in the ARE-treated dogs compared to that in the placebo control group. In addition, the levels of L-citrulline were significantly modulated by ARE intervention, whereas the intervention did not affect intestinal-type alkaline phosphatase (I-ALP), lactate, and carbamoyl-phosphate synthase (CPS). Interestingly, the faecal score reduced significantly with ARE (*p* < 0.001), while the faecal pH remained unaltered. Compared to the baseline, ARE significantly decreased two microbial metabolites, propionic acid, and total short chain fatty acids (SCFAs) levels after 60 days of intervention, whereas butyrate and acetic acid levels remained unchanged in the faecal samples.

**Conclusion:**

In summary, these findings suggest that ARE has gut health promoting benefits in healthy geriatric dogs by improving haematological and biochemical profiles; the levels of L-citrulline; propionic acid; and SCFA; thus, reducing age-related changes by modulating the microbiome and the associated metabolites.

## 1 Introduction

Undoubtedly, the gut microbiome has emerged as a new virtual organ which is crucially important for host homeostasis with profound roles played in different age groups (1, 2). The gut microbiome plays critically essential roles in nutrient assimilation and vitamin absorption and has a vital function in regulating the immune system (3). Gut health is influenced by numerous factors such as environment, nutrition, anxiety disorders, and other confounding factors (4). The developments in the area of gut microbiome are also initiating new research questions in canine medicine and the possible implications in clinical practice. Furthermore, the gut microbiome has been validated to communicate with the brain which is known as the gut–brain axis (5). This communication is of paramount importance for gaining insights into healthy ageing and how modulation of the gut microbiome may aid in achieving healthy ageing goals in geriatric canine patients (6, 7).

Amongst various health issues dogs face, general anxiety in geriatric dogs is a significant challenge as it poses challenging diagnostic hurdles for the clinician (8). This is of great concern as anxiety can trigger a cascade of various other health ailments, and may reduce the life span of the dog and is an area for extensive investigation especially in ageing dogs (9). Studies about the role of the gut microbiome on anxiety disorders are well established in humans; however, there is a lack of similar investigations in canine medicine (10). Based on the data about the role of the gut microbiome in behavioral science in human medicine, it may prove to be pivotal in canine medicine as well. Furthermore, there is an interplay of humoral, metabolic, nervine, and hormonal mechanisms to fully understand the role of the gut microbiome on behavior in dogs which raises a serious concern due to limited available data (11–13).

Herbal supplements are used throughout the globe for their health-promoting benefits, and numerous products have shown interesting effects on the gut microbiome (14, 15). Thus, rational modulation of gut microbiota with suitable herbal supplements may improve the gut health and, in turn, favor the neurobehavioral and physiological responses of the animals.

Ashwagandha, also known as Winter cherry (*Withania somnifera*), is a well-known herbal medicine used in alternative medicine. It is known to provide health-promoting benefits owing to its potent anti-inflammatory, anxiolytic, antimicrobial, and adaptogenic properties mainly due to the presence of withanolides such as withaferin A (16–22). Ashwagandha root extract (ARE) is the most effective form for desired pharmacological effects and its use is gaining wide acceptance in the veterinary practice (23). The general wellbeing of geriatric dogs is essential for pet parents to allow the graceful ageing of their pets. Gastroenterological diseases increase as the dogs grow old which are observed as diarrhoea, inflammatory bowel disease, and colitis. In a study conducted in geriatric canines, Ashwagandha root extract (ARE) supplementation demonstrated hepatoprotective, antiperoxidative, and antioxidant effects (24). In a recent trial, ARE was found to reduce stress in geriatric dogs (25).

The current study evaluated the pharmacological effect of ARE on the gut health of geriatric beagle dogs in a 2-month-long double-blinded placebo-controlled randomized clinical trial. We hypothesized that ARE, by virtue of its antioxidant, anti-inflammatory, and immunomodulatory effects, may improve gut integrity and facilitate a healthy microbiome. The haematological profile and biochemical estimation of liver and kidney function were conducted to investigate this research question. The lipid profile of dogs was evaluated at three time points. Furthermore, the plasma estimation of vital gut integrity markers such as lactate, carbamoyl-phosphate synthase (ammonia), intestinal-type alkaline phosphatase (I-ALP), and L-citrulline was carried out. Furthermore, the faecal scoring, pH, and fat content were observed. In addition, the effect of ARE on microbial metabolites was probed by gas chromatography Fourier transform infrared spectrometry (GC-FTIR)-based mechanistic studies.

## 2 Materials and methods

### 2.1 Chemicals and reagents

The study was conducted using high withanolides concentration (>5%) enriched KSM-66 Ashwagandha root extract (ARE) and corn starch (placebo) which were provided as gift samples by Ixoreal BioMed, Inc., Los Angeles, CA, United States. The chemicals used in the study were of analytical grade unless otherwise indicated and were purchased from Sigma–Aldrich, MA, USA. The enzyme-linked immunosorbent assay (ELISA) kits for canine lactate (CK-Bio-27475), canine carbamoyl-phosphate synthase (ammonia), mitochondrial (CPS1) (CK-Bio-25771), canine intestinal-type alkaline phosphatase (I-ALP) (CK-Bio-27476), and canine citrulline (CK-Bio-27477) were purchased from Shanghai Coon Koon Biotech Co., Ltd. Shanghai, Municipality of Shanghai, China

### 2.2 Study design

The double-blind placebo-controlled trial was conducted in a randomized manner. The study was approved by the local Institutional Animal Ethics Committee (IAEC) and the central government’s Committee for Control and Supervision of Experiments on Animals (CCSEA), New Delhi, India, vide approval number: 03/LA/CVSC-WGL/2023. Throughout the study, dogs were humanely treated with access to commercial pelleted (Pedigree, Mars Inc., USA) food and water. The handling procedures were performed by trained technicians with minimal stress. An expert veterinarian regularly observed the animals for any clinical signs of distress.

### 2.3 Study animals and trial interventions

The study was conducted on 12–15-year-old beagle dogs bred for research purposes (*n* = 12) weighing approximately 11 kg at study initiation which were randomly divided into placebo (*n* = 6) and ARE (*n* = 6) groups. The dogs were clinically healthy, devoid of any signs of any dermatological and neuromuscular disorders. The study was conducted at a certified kennel in Hyderabad, Telangana, India. The dogs were housed in a hygienic kennel at ambient temperature (22–24°C) and were provided with food and water ad libitum. ARE and the placebo products were packed into identical capsules to ensure blinding. All study dogs were assessed on day 0 (study initiation), on days 30 and 60. The placebo group received starch-filled capsules identical to ARE’s appearance, color, odor and taste (KSM-66). The study dogs received either ARE or placebo in the form of capsules daily once, orally at a dose of 15 mg/kg body weight. The dose selection was based on published literature (23).

### 2.4 Faecal sample extraction for faecal metabolite analysis

The measurement of faecal metabolites such as acetic acid, propionic acid, butyric acid and total short-chain fatty acids was performed by GC-FTIR as per the established protocol. Briefly, faecal samples weighing about 1 g were transferred into a separate 50-ml centrifuge tube and mixed with 40 mL of ethanol: water (90:10) mixture. The samples were shaken well by using an orbital shaker for 2 h. After 2 h, samples were centrifuged at 4,000 rpm for 10 min, and the sample solutions were individually transferred into 250 mL bottles and were labelled. The above extraction procedures were performed for each sample, and the solutions were individually transferred into respective tubes. Finally, 2-μl-pooled samples were analysed using the GC-FTIR method. The quantification was performed on a Shimadzu Nexis GC FID-2030 system, Shimdzu Corporation, Japan with an HP-FFAP column and a flame ionization detector. The column flow was maintained at 2.5 mL/min, and the total run time was 23.3 min.

### 2.5 Haematological analysis and biochemical estimations

The blood profile of the study animals was studied using a haematological analyser for RBC count, haemoglobin, and differential leukocyte count. Furthermore, animal plasma was subjected to biochemical analysis for glucose, total protein, globulin, calcium and phosphorus using commercially available kits. Liver function was estimated by measuring total bilirubin, cholesterol, high-density lipoprotein (HDL), low-density lipoprotein (LDL), ALT, AST, and alkaline phosphatase (ALP) and urea and creatinine in plasma for the kidney using commercial kits.

### 2.6 Measurement of plasma levels of important gut health markers

Estimating gut health markers in plasma is critical to assess the impact of pharmacological intervention. To this end, the levels of intestinal type ALP, lactate, and carbamoyl-phosphate synthase (ammonia)—mitochondrial (CPS1) and L-citrulline—were measured by ELISA. Briefly, the samples and standard were added (50 μL) to precoated plates, followed by the addition of 50–100 μL of HRP conjugated reagent. The plates were gently shaken on an orbital shaker and incubated for 60 min at 37°C. After incubation, plates were washed 5× with wash buffer, followed by incubation with 50 μL chromogen for 15 min in the dark for color development. The reaction was stopped using stop solution, and the absorbance was measured at 450 nm. The quantification of samples was carried out based on a standard curve equation.

### 2.7 Measurement of faecal score, pH and fat

The faecal score was estimated based on the Purina faecal scoring chart. The faecal pH was measured with a pH meter (1:10 diluted faeces in distilled water) to assess the effect on the overall acidity/basicity of the faeces. The faecal fat was measured to determine if the stool fat was in the normal range or not.

### 2.8 Statistical analysis

The results were compared between ARE and placebo groups over time, and the data are represented as mean ± standard error of the mean (SEM). All the results between groups were assessed by two-way analysis of variance (ANOVA) followed by Bonferroni’s *post hoc* analysis. The effects of the intervention were evaluated based on *p* < 0.05 for statistical significance. GraphPad Prism version 8.0, GraphPad Software, Inc., USA was used for statistical methodology.

## 3 Results

### 3.1 Effect of ARE intervention on blood parameters

**Figure 1:**
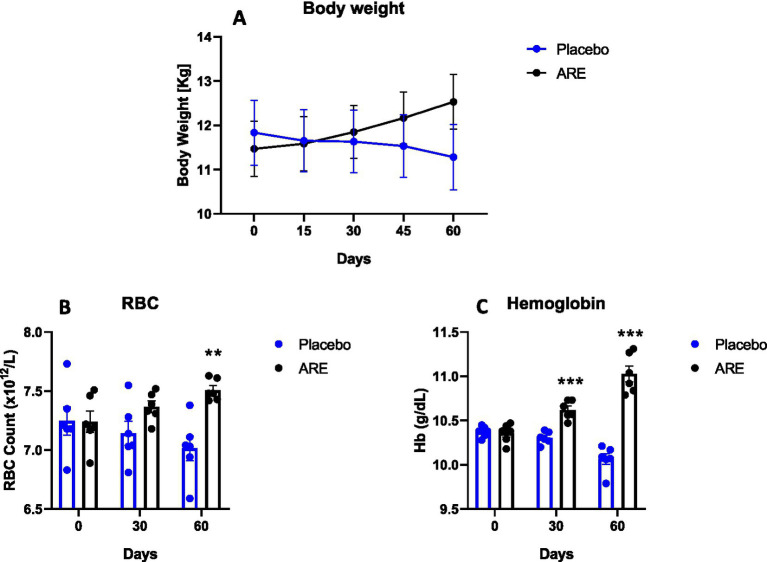
Effect of Ashwagandha root extract (ARE) and placebo treatment on body weight and haematological parameters of beagle dogs treated for 60 days. **(A)** Body weight; **(B)** red blood cell count (RBC), and **(C)** haemoglobin (Hb). Data *n* = 6; statistically analysed by mean ± standard error of the mean (SEM). **Significantly different from placebo group at *p* < 0.01; ***Significantly different from placebo group at *p* < 0.001.

**Figure 2:**
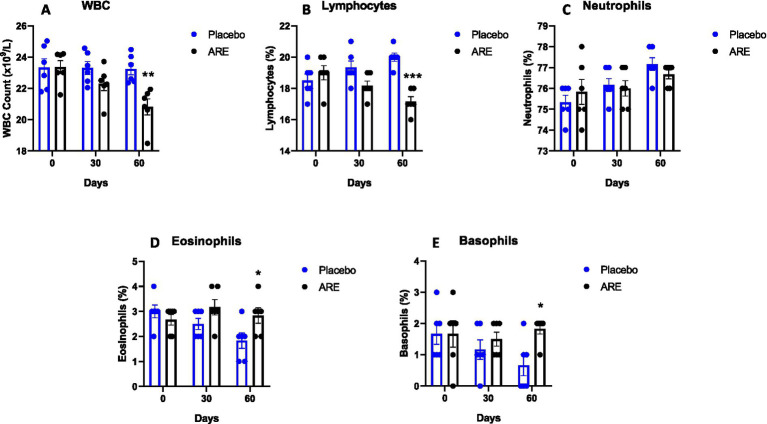
Effect of Ashwagandha root extract (ARE) and placebo treatment on differential leukocyte count of beagle dogs treated for 60 days. **(A)** White blood cells (WBC), and **(B)** lymphocytes, **(C)** neutrophils, **(D)** eosinophils, and **(E)** basophils. Data *n* = 6; statistically analysed by mean ± standard error of the mean (SEM). *Significantly different from placebo group at *p* < 0.05; **Significantly different from placebo group at *p* < 0.01; ***Significantly different from placebo group at *p* < 0.001.

The animal body weights were monitored after every 15 days until study completion. Interestingly, the body weight of dogs treated with ARE showed a trend of increase after 2 weeks of intervention onwards (Figure 1A). However, the changes were not statistically significant. The effect of ARE and placebo intervention on haematological parameters was assessed. The RBC levels were statistically higher in ARE-treated dogs compared to the placebo group on day 60 with *p* < 0.01 (Figure 1B). The haemoglobin levels were observed to be higher in the ARE-treated dogs after 30 days of treatment (*p* < 0.001 vs. placebo; Figure 1C) and 60 days of treatment (*p* < 0.001 vs. placebo; Figure 1C). The parameters of differential leukocytes were assessed to understand the effect of ARE intervention on white blood cell (WBC) dynamics. The WBC count was statistically lower in the ARE-treated dogs after 60 days compared to the placebo group (*p* < 0.01 vs. placebo; Figure 2A). The lymphocyte count was significantly lower in the ARE-treated group compared to placebo after 60 days (*p* < 0.001 vs. placebo; Figure 2B). The neutrophil count was observed to be unaltered in both the study groups throughout the study duration (Figure 2C). The eosinophil count was observed to be significantly higher in ARE-treated group compared to the placebo group after 60 days (*p* < 0.05 vs. placebo; Figure 2D). Similarly, the basophil count was found to be statistically higher in the ARE-treated dogs compared to the placebo control group (*p* < 0.05 vs. placebo; Figure 2E).

### 3.2 Effect of ARE intervention on biochemical parameters of kidney and liver function

**Figure 3:**
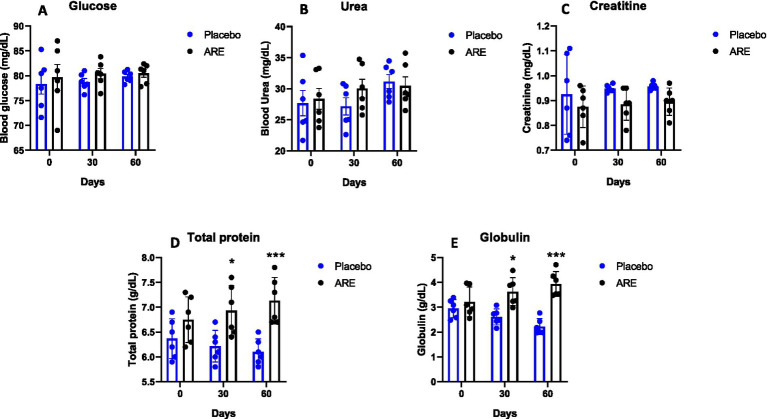
Effect of Ashwagandha root extract (ARE) and placebo treatment on plasma biochemical parameters of beagle dogs treated for 60 days. **(A)** Glucose, **(B)** urea, **(C)** creatinine, **(D)** total protein, and **(E)** globulin. Data *n* = 6; statistically analysed by mean ± standard error of the mean (SEM). *Significantly different from placebo group at *p* < 0.05; ***Significantly different from placebo group at *p* < 0.001.

**Figure 4:**
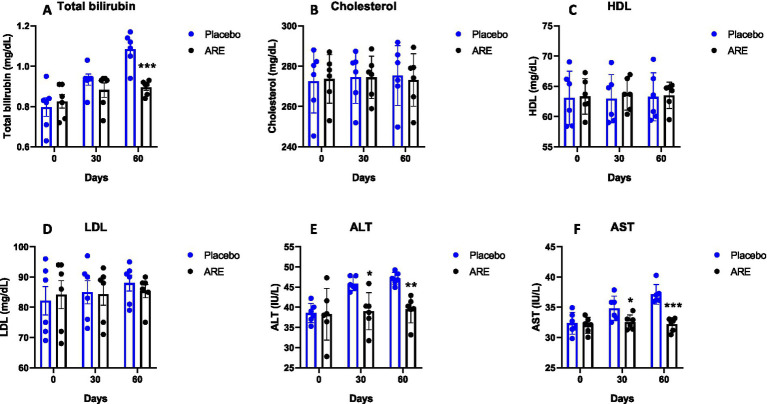
Effect of Ashwagandha root extract (ARE) and placebo treatment on plasma biochemical parameters of beagle dogs treated for 60 days. **(A)** Total bilirubin, **(B)** cholesterol, **(C)** HDL, **(D)** LDL, **(E)** ALT, and **(F)** AST. Data *n* = 6; statistically analysed by mean ± standard error of the mean (SEM). *Significantly different from placebo group at *p* < 0.05; **Significantly different from placebo group at *p* < 0.01; ***Significantly different from placebo group at *p* < 0.001.

Glucose levels were unaltered in both the study groups (Figure 3A). Urea and creatinine are essential kidney function markers and were unaltered after ARE treatment, indicating no alteration of normal renal physiology (Figures 3B,C). The quantity of total protein and globulin were observed to be statistically higher in the ARE-treated group compared to the placebo control group (*p* < 0.05 vs. placebo at 30 days for both the markers, *p* < 0.001 vs. placebo at 60 days for both the markers; Figures 3D,E). The quantity of total bilirubin was found to be significantly lower after 60 days in the ARE-treated dogs compared to the placebo control group (*p* < 0.001 vs. placebo; Figure 4A).

**Figure 5:**
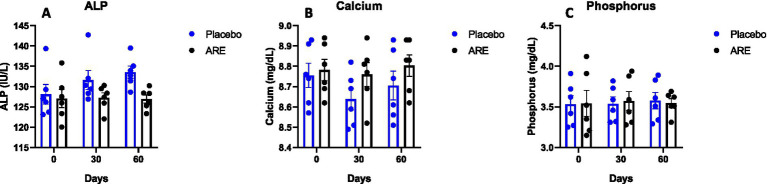
Effect of Ashwagandha root extract (ARE) and placebo treatment on plasma biochemical parameters of beagle dogs treated for 60 days. **(A)** ALP, **(B)** calcium, and **(C)** phosphorus. Data *n* = 6; statistically analysed by mean ± SEM.

The quantity of cholesterol, HDL, and LDL were measured. No alteration in the quantity of these three lipid markers indicated the safety of ARE (Figures 4B–D). The quantity of ALT enzyme was observed to be significantly lower in ARE-treated dogs after 30 (*p* < 0.05 vs. placebo; Figure 4E) and 60 days (*p* < 0.01 vs. placebo; Figure 4F). Furthermore, the quantity of AST enzyme also showed a similar pattern with statistical significance after 30 (*p* < 0.05 vs. placebo; Figure 4F) and 60 days (*p* < 0.001 vs. placebo; Figure 4F). Importantly, all the values were within the physiological range. The ALP values also showed a pattern of decline with ARE treatment compared to the placebo group. However, the results were not statistically significant (Figure 5A).

### 3.3 Effect of ARE intervention on calcium and phosphorus

The plasma levels of calcium and phosphorus were unaltered after 2 months of treatment of ARE (Figures 5B,C), demonstrating that the intervention is safe and does not perturb the physiological mineral balance.

### 3.4 Effect of ARE intervention on plasma markers of gut health

**Figure 6:**
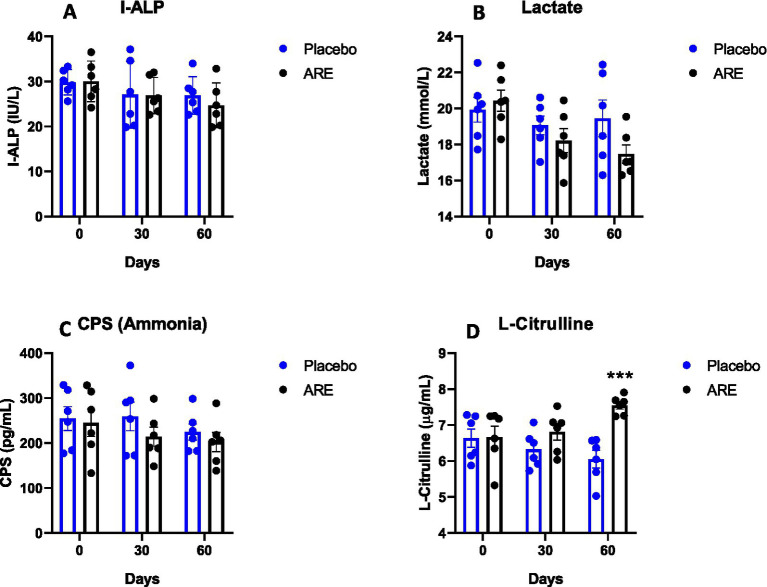
Effect of Ashwagandha root extract (ARE) and placebo treatment on plasma parameters of gut integrity of beagle dogs treated for 60 days. **(A)** Intestinal-type alkaline phosphatase (I-ALP), **(B)** lactate, **(C)** carbamoyl-phosphate synthase (CPS), and **(D)** L-citrulline. Data *n* = 6; statistically analysed by mean ± standard error of the mean (SEM). ***Significantly different from the placebo group at *p* < 0.001.

To assess the impact of ARE on gut health, the levels of I-ALP, lactate, CPS, and L-citrulline were measured in the plasma. The levels of I-ALP showed a trend of decline in the ARE-treated dogs’ plasma compared to the placebo after 60 days of treatment (Figure 6A). However, the results were not statistically significant. The lactate values also exhibited an interesting trend of decline upon ARE treatment from 30 days onwards, and a similar trend was observed after 60 days (Figure 6B). However, the trend in reduction was not statistically significant and all the values remained in physiological ranges. The quantity of CPS (ammonia) was observed to be inhibited by ARE intervention as compared to the placebo control group; however, the decline was not statistically significant (Figure 6C). The levels of L-citrulline indicate intestinal integrity and homeostasis. The quantity of L-citrulline was significantly higher in ARE after 60 days of treatment compared to the placebo control group (*p* < 0.001 vs. placebo; Figure 6D).

### 3.5 Effect of ARE intervention on important faecal parameters

**Figure 7:**
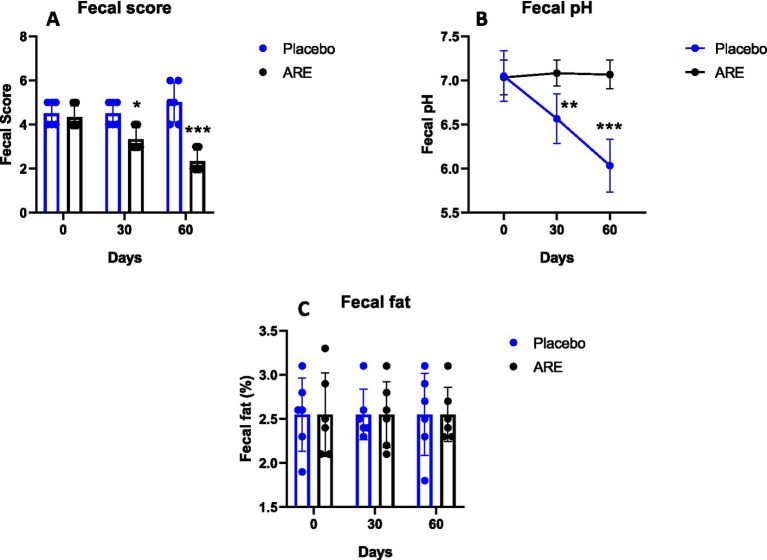
Effect of Ashwagandha root extract (ARE) and placebo treatment on faecal parameters of beagle dogs treated for 60 days. **(A)** Faecal score, **(B)** faecal pH, and **(C)** faecal fat. Data *n* = 6; statistically analysed by mean ± standard error of the mean (SEM). *Significantly different from placebo group at *p* < 0.05, **Significantly different from placebo group at *p* < 0.01; ***Significantly different from placebo group at *p* < 0.001.

The faecal score indicated that ARE plays a role in the reduction as it was observed to be significantly lower in ARE-treated group compared to the placebo control group after 30 (*p* < 0.05 vs. placebo; Figure 7A) and 60 days (*p* < 0.001 vs. placebo; Figure 7A). ARE intervention does not lead to a significant reduction in faecal pH, whereas reduction in the faecal pH was seen in placebo control group after 30 (*p* < 0.01 vs. ARE; Figure 7B) and 60 days (*p* < 0.001 vs. ARE; Figure 7B) of treatment. Furthermore, the evaluation of faecal fat indicated that it was unaltered in ARE-treated dogs compared to the placebo control group animals (Figure 7C).

### 3.6 Effect of ARE on microbial metabolites important for gut health

**Figure 8:**
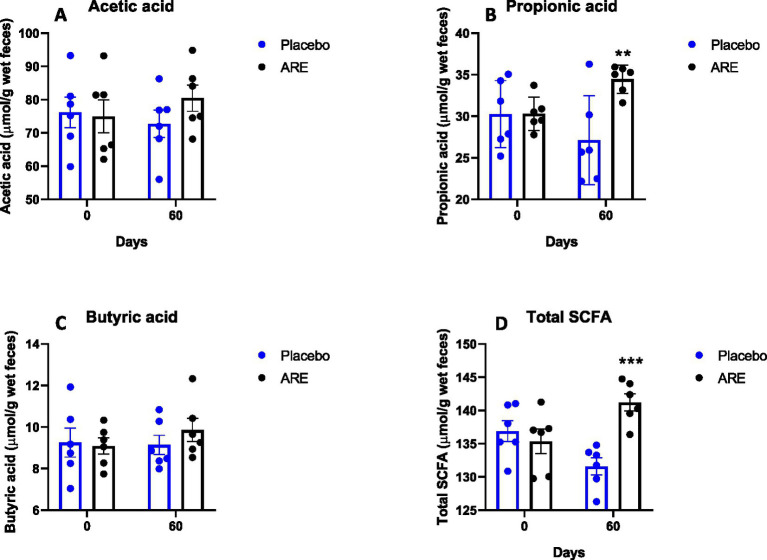
Effect of Ashwagandha root extract (ARE) and placebo treatment on faecal microbial metabolites of beagle dogs treated for 60 days. **(A)** Acetic acid, **(B)** propionic acid, **(C)** butyric acid, and **(D)** total short chain fatty acids (total SCFA). Data *n* = 6; statistically analysed by mean ± standard error of the mean (SEM). **Significantly different from placebo group at *p* < 0.01; ***Significantly different from placebo group at *p* < 0.001.

The levels of acetic acid showed a slight increase after 60 days of ARE treatment; however, the results were not statistically significant (Figure 8A). Propionic acid was significantly higher in the ARE-treated group after 60 days of treatment (*p* < 0.01 vs. placebo; Figure 8B). The quantity of butyric acid was observed to be increasing with ARE intervention; however, the results were not statistically significant (Figure 8C). On the contrary, the levels of total SCFA were statistically higher in ARE-treated dogs after 60 days of treatment (*p* < 0.001 vs. placebo; Figure 8B).

## 4 Discussion

There is a considerable interest in establishing methods to improve the overall wellbeing of pet dogs through the use of nutritional supplements (26). An essential aspect of general health is a healthy well-balanced gut microbiome, which is largely appreciated as the new virtual organ. There is ample evidence in human medicine and emerging veterinary research that gut microbiome plays a critical role in physiological and altered pathological conditions (27, 28). The perturbation of a healthy microbiome can lead to various disease conditions, may trigger specific pathologies, or may aggravate certain underlying diseases (3, 29). Hence, it is important to diligently dissect and understand the role that gut microbiome may play in multiple systemic conditions (30). In the current study, the pharmacological effects of ARE (15 mg/kg) were investigated in geriatric Beagle dogs to dissect its role in the modulation of gut microbiome-relevant parameters. The animal body weight, haematology, and critical organ function parameters were investigated. The plasma markers of a healthy gut, faecal parameters, and microbial metabolites were investigated to obtain hitherto non-available insights into the role of ARE on the canine gut microbiome. The study provides new insights into the promising role of ARE in promoting a healthy gut. Oral dosing of ARE for 2 months led to improved physiological state, organ function, and beneficial effects on the microbiome.

There was a trend of an increase in the body weight of ARE-treated animals. We did not measure the diet intake of animals during the study. It might be possible that the ARE-increased appetite of treated animals leads to increased body weight, which in turn may be responsible for altering other biochemical parameters. Interestingly, the levels of RBCs and Hb were found to be significantly higher after ARE intervention. These findings directly correlate with several studies indicating that ARE potentially acts as a haematopoietic (31). On the contrary, the levels of WBC were lower in ARE-treated dogs while maintaining all the values within the physiological range.

In the case of geriatric dogs’ assessment of liver and renal functions is critical which plays an important role in metabolism and clearance, including glucose (32, 33). In the study, we observed that glucose levels were well within the physiological range after 2 months of intervention. Interestingly, the renal function markers urea and creatinine remained in a healthy physiological range throughout the study duration. Of note, the levels of total protein and globulin were found to be significantly high in ARE-treated dogs. The lipid levels are maintained within normal ranges primarily by the metabolic function of the liver (34). The HDL and LDL cholesterol levels were within safe physiological range after 2 months of treatment with ARE and placebo. ALT and AST levels were observed to be lower in ARE-treated animals compared to the placebo control group and remained within the physiological range.

The levels of inorganic minerals, especially calcium and phosphorus, are critically important for muscle function and enzymatic reactions (35, 36). Lowered calcium and phosphorus levels may indicate reduced bone strength, affecting healthy ageing (37). Herein, calcium and phosphorus levels remained unaltered, showing that are ARE intervention is safe to use and does not alter Ca/P homeostasis throughout the trial.

I-ALP is a well-known anti-inflammatory enzyme produced by small intestine enterocytes and is secreted into the lumen, blood, and stool (38). I-ALP aids in reducing the toxicity of LPS by two mechanisms: by removing one of the phosphate moieties resulting in the generation of monophosphate LPS or by detoxification of nucleotides such as adenosine triphosphate (ATP) and uridine triphosphate (UDP) by dephosphorylation (39). I-ALP can elicit autophagy-dependent anti-inflammatory function using inhibiting the LPS-induced inflammatory NFκB-p65 phosphorylation along with the IL-1β gene expression in macrophages. It has a possible role in mucin production as well (40). We did not observe any modulation of I-ALP by ARE throughout the duration of the study. Any change in gut health is indicated by a rise in I-ALP in faeces and blood. A non-significant decline shows that the enterocyte villus membrane at the site of I-ALP was unaffected. Furthermore, lactate is also a critical gut health parameter. It is produced from lactic acid bacteria and acts as a signaling molecule through G-protein-coupled receptor 81 (GPR81). Under normal conditions, the gut membrane is impermeable to the lactic acid produced, and the enzymes rapidly disintegrate a small quantity of lactic acid which rarely escapes into the circulation. However, intestinal permeability is enhanced during gut-dysbiosis, and lactic acid may enter the circulation. Higher lactic acid levels may result in acidosis in rare cases, affecting normal brain functions and may decrease the brain pH thereby affecting the secretion of neurotransmitters (41). Hence, it is a good choice to be called a gut health biomarker. The intervention modulated the levels of lactate. In addition, the levels of CPS (ammonia) were unaltered throughout the duration of the study. Interestingly, the levels of L-citrulline were significantly reduced by ARE intervention, an essential marker for maintaining a good gut microbiome.

The stool parameters are essential in assessing gut health (28, 42). The faecal score was significantly lower in ARE-treated dogs, strongly indicating improved stool quality. Furthermore, placebo dogs showed a decline in faecal pH, whereas the stool pH of ARE-treated dogs remained unaltered, indicating no change in acidic microbial metabolites. The reduction in the faecal pH of control dogs was probably due to interference by starch which has been earlier shown to reduce faecal pH amongst dogs, pigs, and cows (43–45). This finding suggests that starch may not be an appropriate placebo when studying the effects of the intervention on physicochemical features of faeces. Furthermore, the faecal fat remained well within physiological ranges. Interestingly, ARE led to a significant increase in the levels of two important gut metabolites, propionic acid and SCFA which are known to have anti-inflammatory and gut barrier-promoting activities. These results provide great insights into the potential health benefits of ARE for the improvement of geriatric dog gut health, and it may be recommended for use as a health supplement to promote the wellbeing of geriatric and healthy dogs.

The present study also had some limitations which warrant consideration for future studies, including a relatively small sample size and the use of only a single extract of Ashwagandha. On the contrary, we did not measure any behavioral endpoints for the current study, and the detailed sequencing of the microbiome may be envisioned for future studies. In addition, the weight gain in the control group observed could not be delineated in detail as we did not measure the feed intake; however, it might possibly be due to enhanced appetite by ARE or increased palatability of ARE-containing diet which requires investigations. Furthermore, the effects of starch on faecal pH and possibly on microbiome show that starch may not be a good candidate to be used as a placebo in studies investigating the effect of the intervention on faeces.

## 5 Conclusion

The results of the current study provide insights into the beneficial role of ARE in the modulation of gut health in geriatric dogs. ARE modulated the gut mechanistically by modulation of L-citrulline, whereas I-ALP, lactate, and CPS remained unchanged. Interestingly, ARE modulated the faecal score without altering the faecal pH. In addition, this study sheds light on the possible role of propionic acid and total SCFA behind the proposed anti-inflammatory effects of ARE and may potentially be of clinical significance for improved gut health and its implications on geriatric dogs. Detailed investigations are needed in the future to gain a deeper understanding of the modulation of the gut microbiome by ARE.

## Data Availability

The raw data supporting the conclusions of this article will be made available by the authors, without undue reservation.
